# A concept for adapting medical education to the next generations via three-staged digital peer teaching key feature cases

**DOI:** 10.1007/s10354-022-00990-7

**Published:** 2022-12-21

**Authors:** Paul Supper, Damaris Urban, Iris Acker, Florian Simon Linke, Patric Kienast, Andrea Praschinger, Anahit Anvari-Pirsch

**Affiliations:** 1 Teaching Center, Medical University of Vienna, Vienna, Austria; 2 Clinical Department of Plastic and Reconstructive Surgery, Medical University of Vienna, Waehringer Guertel, Vienna, Austria; 3 Klinische Abteilung für Neuroradiologie und Muskuloskeletale Radiologie, Universitätsklinik für Radiologie und Nuklearmedizin, Vienna, Austria

**Keywords:** Generation Z, Medical education, Peer teaching, Key feature cases, Interactive teaching, Generation Z, Medizindidaktik, Peer-Teaching, Key-Feature-Fälle, Interaktives Unterrichten

## Abstract

While the core principles of medical education remain the same, the students’ socioecological backgrounds, values and learning requirements are constantly changing. Bridging the generation gap between teachers and students is a key challenge of medical didactics. To meet the demands of today’s classroom, we piloted a novel three-stage peer teaching and key feature concept. First, an on-demand key feature video case was presented. Second a background video was launched, followed by a self-assessment tool. Third, a live case discussion webinar focusing on clinical reasoning was held. The contents were created by near-peers experienced in medical didactics and checked by clinical experts. The elective format resonated with 652 participating graduate students and 1250 interactions per webinar, suggesting that students’ strengths and weaknesses were addressed adequately. We aim to provide educators with input for creating a flexible and integrative learning environment utilising modern technological and didactic tools that shape the healthcare workers of tomorrow.

## Abstract (de)

Während die Grundprinzipien der medizinischen Ausbildung gleichbleiben, ändern sich der sozioökologische Hintergrund, die Werte und die Lernanforderungen der Studierenden ständig. Die Überbrückung der Generationskluft zwischen Lehrenden und Lernenden ist eine zentrale Herausforderung der Medizindidaktik. Um den Anforderungen des heutigen Unterrichts gerecht zu werden, haben die Autoren ein neuartiges 3‑stufiges Peer-Teaching- und Key-Feature-Konzept erprobt. Zuerst wurde ein On-Demand-Key-Feature-Fall präsentiert. Anschließend wurde ein Hintergrundvideo mit anschließender Selbstüberprüfung freigeschaltet. Zuletzt wurde eine Online-Live-Falldiskussion abgehalten, welche sich auf klinische Argumentation konzentrierte. Die Inhalte wurden von medizindidaktisch geschulten Near-Peers erstellt und von klinischen Experten überprüft. Das Format des Wahlfachs fand bei 652 teilnehmenden Studierenden und mit 1250 Interaktionen pro Webinar großen Anklang, was darauf schließen lässt, dass die Stärken und Schwächen der Studierenden adäquat angesprochen wurden. Ziel der Autoren ist es, Lehrkräften Anregungen für die Schaffung einer flexiblen und integrativen Lernumgebung mittels moderner technologischer und medizindidaktischer Tools zu geben, um das Gesundheitspersonal von morgen auszubilden.

## Introduction

### Generation gap between practicing teachers and millennial students

Bridging the generation gap is a known issue in medical education [1, 2]. Nowadays, the percentage of students belonging to the so-called Generation Z (born between 1995 and 2010) is increasing. Generation Z students grew up in a digital era characterised by extraordinarily rapid technological progress reaching private households, resulting in an extensive usage of as well as exposure to digital media [3, 4]. Along with all the conveniences this technological advancement brought, parents, students and teachers were also faced with many new challenges. Generation Z students often seem to be inadequately reached by the frontal teaching of a traditional lecture.

Educators, most of whom are members of the baby boomer generation, experienced their education mainly based on lectures and textbooks, while Generation Z prefer web-based didactic methods, video-based presentation of learning materials, story-telling and immediate feedback for self-directed learning [5–7]. Teachers are confronted with adapting their didactic methods to keep their students engaged in learning. Reyes et al. [8] showed that self-description as competence-centred was a common characteristic for teachers of the X, Y and Z generations. Health teachers of Generation Y were characterised by empathetic teaching, whereas educators of the Generation Z were branded as innovative [8]. While the usage of web-based media is high, it is only of minor importance in medical didactics, most likely due to the requirement of increased efforts as well as lack of support for medical teachers to reach the wide range of fast-changing digital tools [9]. Peer teaching can assist in filling the gap of lacking support for using digital tools for medical teachers. On the other hand, peers profit from experienced medical teachers, and working together results in a productive synergy for teaching complex medical issues. Generation Z has a high affinity for web-derived information and is more accustomed to multitasking, but critical thinking might be lacking, especially in the differentiation between opinions and facts [3, 10]. Shatto et al. [10] postulate that bridging the generational gap can be achieved by adapting to modern methods, while creating a dynamic learning environment with more interactions to meet the students’ learning requirements.

### Requirements for teaching clinical reasoning to Millennials

The importance of teaching critical thinking in medicine is well known and has been addressed for a long time, as it is crucial for clinical reasoning [11–15]. Due to their expertise and incorporation of their applied clinical knowledge, experienced physicians might focus their teaching on detailed and complex issues, overlooking students’ difficulties in completing the fundamental tasks of clinical reasoning, including forming differential diagnoses or assessing the urgency of the clinical situation. Nonetheless, although these challenges were always present in medical education, methods of teaching need to adjust to optimally reach Generation Z. Reaching Generation Z students is possible by adjusting the teaching methods.

Not only have the socioecological and learning modalities of students changed, also the working and knowledge requirements at clinical entry seem to rise continuously. In order to equip students of the new generation with the appropriate knowledge, skills and competencies to meet their increasing requirements, it is also necessary to continuously optimise their medical training. Despite previously acquired theoretical fundamentals of clinical workflows, millennial students entering clinical practice are often confronted with clinical situations beyond their capabilities.

A central element of clinical work is forming a diagnosis, which is prone to underlying cognitive biases, which makes up over 70% of diagnostic errors [13, 16–18]. Reflection and critical thinking are crucial elements for preventing and debugging cognitive biases. These two cognitive skills need to be strengthened in Millennials, as the flood of today’s information on social media tempts one to 1) jump to quick conclusions (availability bias), 2) persist with one’s ideas (anchoring bias, overconfidence bias) and 3) promotes selective perception of matching information (confirmation bias). Teaching micro-skills for critical thinking that prevent those thinking schemes allows medical educators to strengthen clinical reasoning in millennial students [13]. Specific methods to incorporate this into lessons are case simulation, active questioning, stimulating reflection and discussion and creating a framework that addresses problem-solving and decision-making [11, 12].

## Methods

This course was implemented as an elective for emergency medicine, gynaecology, ophthalmology, otorhinolaryngology, neurology, paediatrics and psychiatry, where students could choose between different formats to finish the course. The cases were created by four near-peers who had already experienced the clinical transition and were trained in medical didactics, who each presented and discussed clinical cases in three stages: a key feature video presentation, a remote background presentation and an interactive live case discussion.

### Three-stage key feature cases

In the first stage, peers presented one of their key feature cases [19] as an on-demand 20-minute pre-recorded video with embedded interactive elements (e.g., immediate questions, hint fields, links to previous chapters). While watching the case presentation, the students were questioned regarding urgency, differential working diagnosis, speciality-specific history, physical examination and further diagnostic examinations, i.e., imaging modalities and other specialised tests.

The second stage was an on-demand video presentation revising previous learning materials and presenting guidelines and current literature to explain the case’s background (e.g., aetiology, diagnostic criteria, state-of-the-art treatment). Direct links to further learning materials were provided for individual in-depth study, including links to the corresponding resources, such as items in the main curriculum, current guidelines and recent scientific papers. To keep the level of student engagement elevated, the background videos were limited to a maximum time of 10 min. This stage was closed by a short self-assessment in the form of multiple-choice questionaries and drag-and-drop exercises among other tasks.

The third stage was the interactive case discussion. This 45-minute live webinar comprised a discussion between students and moderating peers to stimulate reflection focusing on clinical decision-making. The webinar was recorded and made available for review on the learning platform of the university (Table 1).

**Table 1:** Concept of three-staged digital peer teaching key feature cases: the first and second stages are provided as on-demand contents via the learning platform, while the case discussion is held as a live webinar

Stage	Content
*Moodle on-demand content*	
1) Key feature case	Video presentation simulating clinical competence and stimulating reflection
2) Remote background	Video presentation of previous learning materials, relevant guidelines and recent publications
Self-assessment	Questionnaires and exercises
Feedback	One-minute paper
*Live webinar*	
3) Case discussion	Discussion and reflection focusing on clinical decision-making

### Case development and quality control

Instruments for longitudinal evaluation were used for continuous refinement. This enabled a high level of security in terms of content and medical didactics in peer teaching. The case reports of the peers were reviewed by clinical experts during the clinical practical year. Subsequently, anonymised preparation of the key feature cases and the remote background was conducted, which was checked by the course coordinator and by a tutor regarding correctness of content and comprehensibility. Prior to initial performance, a final review was performed by experienced clinicians. By guiding the near-peers by experienced clinicians and medical teachers, this program ensured the high educational standards expected of a university course were met.

After completion of the on-demand content, the standardised student feedback took place. For this purpose, a one-minute paper was used, which can be answered within 1 min due to the condensed question format and still provides essential feedback. The following three questions were addressed: 1) What was the most essential thing you learned today? 2) What important question remained unanswered? 3) Other comments on the presentations [20]. This compact feedback format allowed quick evaluation, so that the improvements were already applied to the live case discussion. The on-demand contents were launched twice weekly, with 2 days preparation time before the live discussion. All cases were presented in anonymised form. Students were asked to give active consent to the privacy policy. To individually support the students, a frequently asked question (FAQ) section, an e‑mail support and a user-friendly interface including an introductory video were established.

### Technical equipment and human resources

Since our course should be accessible to every student, easy to implement in every university and easy to prepare for peers, using as much pre-existing infrastructure as possible was a main concern when designing this course. Therefore, this course was built upon the institution’s learning platform to host interactive videos, learning resources and self-assessment forms. This comprised the first two stages—the key feature cases and remote background with self-assessment—which were presented via remote access utilising the interactive video H5P (https://h5p.org/) tool. The live webinar was hosted by a contracted third-party provider for videoconferencing in order to spare institutional technical resources. The course was supported by paid student tutors who engaged in video editing and upload, technical implementation of the interactive H5P elements, assisting with the live moderation and administrative tasks such as checking attendance. The team of near-peers consisted of 4 students, all of whom had previously received special training concerning didactic principles in the form of workshops and/or employment as a student tutor. Each of the prepared key feature cases written by the peers was originally experienced in their own clinical years.

## Results

### Course participation

**Fig. 1:**
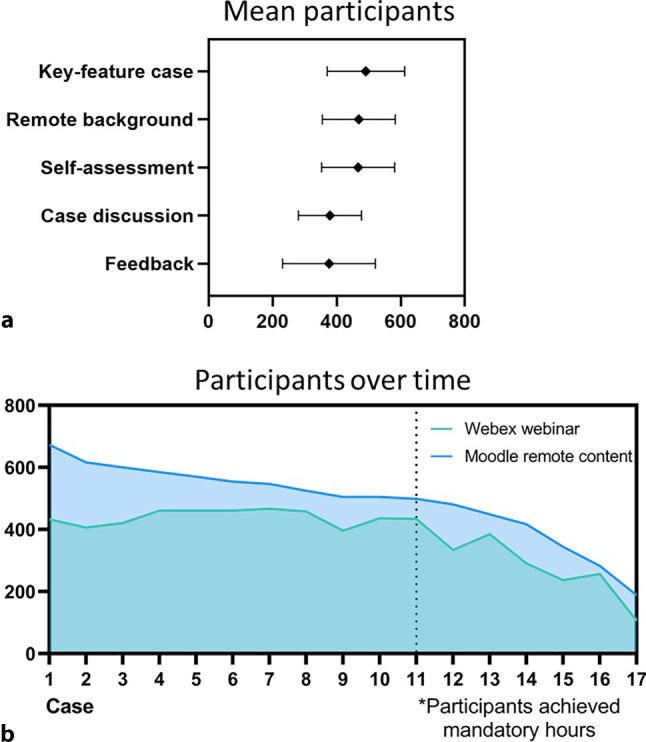
Participation in the three-staged digital peer teaching key feature cases: **a** mean participants in the several elements shown as mean and standard deviation, **b** participation over time

The course was introduced as an elective for emergency medicine, gynaecology, ophthalmology, otorhinolaryngology, neurology, paediatrics and psychiatry. In total, 652 fifth- and sixth-year medical students attended the course. A total of 35 academic hours including 17 interdisciplinary key feature cases with a total of 6 h of interactive on-demand video material were produced. 13.5 h of live webinars were delivered, and 96 advanced-learning sessions were offered. In total, over 139 students enrolled voluntarily and 40% attended more units than they needed to achieve their elective credits (Fig. 1).

### Student interaction

**Fig. 2:**
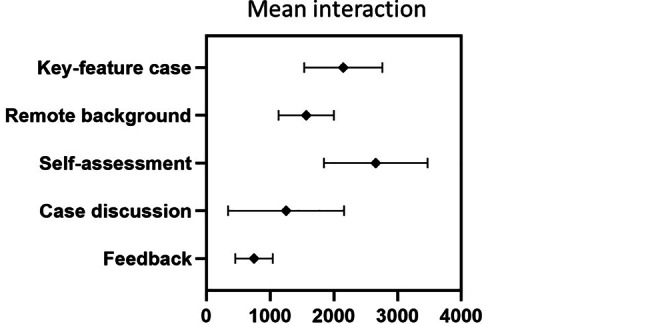
Student interaction in the three-staged digital peer teaching key feature cases: mean interaction in the several elements shown as mean and standard deviation

The on-demand key feature cases had an average participation of 451 students, while the webinars averaged 397 active students. Within the webinar duration of 45 min, an average of 1250 interactions were made by participants through answering questions or sharing reflections in the chat. Overall, on-demand contents generated 120,322 interactions (Fig. 2 and Table 2).

**Table 2:** Student interaction in the three-staged digital peer teaching key feature cases: mean and overall interaction (https://moodle.org/)

Interactions	Mean	Overall
*Moodle on-demand content*		
Key feature case	2148	36,530
Remote background	1567	26,647
Self-assessment	2657	45,181
Feedback	747	11,964
*Live case discussion*	1250	18,751

### One-minute paper

The standardised student feedback through the one-minute paper showed a positive response, which was confirmed by the high number of participants and their interactions. Students indicated that they benefited from the reflection on their clinical workflow.

## Discussion

While the students chose to hold their elective using this course, 139 students enrolled completely voluntarily, without curricular need. After reaching the compulsory hours, a decrease in the number of participants was expected. Nevertheless, it is quite remarkable that despite the goal of reaching the necessary hours, a significant number of 260 students continued to follow the program, indicating that we have induced intrinsic motivation for learning. Overall, the participating students managed to complete 99.2% of the on-demand and live contents within the given timeframe and only 0.8% had to hand in a compensation that resembled a guided essay of a self-experienced case. It seems that when entering the clinical training, students learn to value learning opportunities that facilitate clinical reasoning.

### Approaching Generation Z with gamification, individuality and interaction

The three-staged process generated a learning atmosphere that apparently met the students’ needs. Generation Z values independency, flexibility, individual approaches, active integration and technological approaches. All the above were addressed by our program, as validated by the high interaction. Similar to social media, the students showed their interest by high participation, using the content and their particular high number of interactions. In the on-demand contents—the key feature cases—the students had the possibility to follow the content at their own pace and test themselves in the self-assessment. The self-assessment was provided in various formats that resembled gamification of learning materials with instant visual feedback, increasing the positive learning experience. Overall, students not only could study independently and flexibly, but also enjoy novel technical approaches.

Prepared by the background video and their own reflections during the key feature video, students proactively engaged in the live case discussion webinars. Within the 45 min of webinar, an average of 1250 interactions by answering questions and sharing reflections showed engagement, which would be unthinkable within a traditional frontal lecture. This overwhelming number of interactions was made possible by the possibility of simultaneous and multiple responses in the chat. Emphasis is to be laid on the learning atmosphere that was created, which resembled more the audience joker of “Who wants to be a Millionaire?” rather than a tense examining setting. Certainly, an essential factor for this was the use of peers as teachers.

### Utilising peer teaching to address Millennials

While the on-demand contents provide many benefits such as flexibility, individual learning pace and revision—for students and for teachers—the technical possibilities of recorded videos for simulating interaction and reflections, even with the usage of interactive H5P elements, is limited. Personal interaction is inevitable for teaching clinical reasoning. The transition from medical theory to clinical practice provides many challenges, which can be addressed by near-peers who have already mastered this transition. The concept of peer teaching—by students for students—is increasingly finding its way into curricular design. One advantage of peer teaching is the transfer of knowledge “at eye level,” which leads not only to the transfer of pure knowledge, but also of personal experiences and micro-skills with a coach effect [21]. Peers trained in didactic principles can discuss fears and uncertainties that students face, present experienced events of clinical challenges and stimulate reflection of the students’ process of clinical reasoning in a safe yet guided learning atmosphere. The peers also addressed medical interpersonal components as well as experiences in patient contact and situations in which they made their first clinical decisions under supervision. In this way, the attitudes, skills and abilities that the university has taught so far can be expanded through the experiences of the peers. Addressing the challenges of the students at clinical entry through the peers can simulate an adequate incorporation of basic knowledge and trained skills. However, this horizontal approach via near-peer teaching under supervision by professors could also be adapted to a more vertical knowledge transfer via professors, depending on the requirements of the teaching application of this concept.

### Preserving university learning objectives with adaptation of teaching modalities

While adapting teaching modalities to the needs of the new generation, the curricular structure and defined competences must be maintained. During the second stage—the remote background presentation—the relevant learning materials from previous curricular elements are repeated and official learning materials, guidelines and current publications presented. This combines multiple university goals. By revising previous learning materials, vertical learning progress is enhanced through cross-grade learning while cross-linking basic knowledge with clinical thinking. Furthermore, the introduction to current literature, guidelines and studies, as well as the direct linking for independent further studying, deepens evidence-based and lifelong learning.

The main focus of this course was to avoid an exam-like situation and rather to provide a pure self-assessment without feedback to the case authors. However, an exam could easily be implemented if needed.

### Optimizing hybrid education

This teaching concept supports hybrid education and thus the interweaving of face-to-face teaching with e‑learning. The time-independent on-demand content (the interactive key feature cases, the remote background with self-assessment) supports the flexibility and independent learning of different learning types of students. At the same time, this strategy enables teachers to use the teaching content regularly and repeatedly, with the possibility of continuous improvement under high time flexibility. Through the targeted use of case discussion as live content (online seminar or physical), direct exchange is promoted and it is possible to go into depth on topics that particularly interest the students.

### Three-staged digital key feature cases for facilitating clinical teaching in Millennials

Teaching clinical reasoning and decision-making is a particular challenge in medical curricula. After graduating from medical school, students should be able to apply medical skills, knowledge and attitudes in a setting that presents multiple challenges. The key feature cases are a validated approach to establishing decision-making competence, with a focus on the essential decisions in the treatment process that must be recognised and made in order to treat the patient correctly [19]. Through the use of interactive key feature cases, students take a step back, allowing for a differentiated view without stressors such as time pressure or organisational and interpersonal challenges. This variation of clinical training in a reduced and protected setting is intended to promote reflection on simulated clinical decisions, especially through the activating questions, which is often difficult to achieve in the immediate clinical situation. At the same time, students receive guidance and feedback through a structured three-stage set-up in which they are presented with a case via remote access, receive background information, perform a self-assessment and continue in-depth study using the linked learning materials [15].

## Conclusion

By creating an optimal learning atmosphere, this concept creates high engagement and intends to deepen clinical thinking and clinical integration of previously learned skills. The goal of this new teaching concept is to bridge the generation gap between teachers and students and prepare millennial students for today’s challenges in transition into clinical practice. The concept is characterised by its clear design, feasibility and its optimal usage of hybrid education and thus enables implementation in modern medical curricula at other universities. We aim to provide educators with input for creating a flexible and integrative learning environment utilising modern technological and didactic tools that shape the healthcare workers of tomorrow.
